# Equine maxillofacial intraosseous cystic lesions: a retrospective study of 17 cases

**DOI:** 10.3389/fvets.2025.1644866

**Published:** 2025-09-10

**Authors:** Jennifer L. Kelley, Jennifer E. Rawlinson, Cynthia M. Bell

**Affiliations:** ^1^Department of Veterinary Clinical Sciences, College of Veterinary Medicine, University of Minnesota, St. Paul, MN, United States; ^2^Department of Clinical Sciences, College of Veterinary Medicine and Biomedical Sciences, Colorado State University, Fort Collins, CO, United States; ^3^Specialty Oral Pathology for Animals (SOPA), Geneseo, IL, United States

**Keywords:** horse, head, pseudocyst, mass, mandible, maxilla, cyst, dental

## Abstract

**Introduction:**

Cystic and pseudocystic masses of the equine maxilla and mandible are rare lesions that result in clinically significant morbidity and/or mortality for the patient. Previous literature consists of case reports or case series. Few studies have addressed the variety of cystic lesions and comparative features. The aim of this study was to retrospectively describe the clinical signs, imaging findings, histopathologic diagnoses, treatments, and clinical outcomes for cystic masses in the equine maxilla and mandible.

**Methods:**

Cases were recruited from six sources including a pathology laboratory, universities, and multiple private practices. Inclusion criteria were cystic/cavitated lesions within the maxilla mandible, and/or incisive bones that had a complete medical record that included history, gross appearance of the mass, diagnostic imaging, histopathology report, treatments performed, and clinical outcomes. Primary sinus cysts and teratomas were excluded from the study.

**Results:**

Lesions were identified in 17 patients with 1 patient having multifocal maxillomandibular cystic lesions. The most common lesion location was the body of the mandible. Diagnosis of lesion etiology and type was made by assimilating histopathology with clinical findings and imaging results. The following cysts were diagnosed: dentigerous cyst (6), bone cyst (6), and radicular cyst (3). Two lesions were unclassified, radiolucent inflammatory lesions. Patients were treated surgically with marginal excision and/or aggressive cyst lining debridement for 16/17 cases with rostral mandibulectomy performed in 1/17 cases. Excisional biopsies were performed at the time of definitive surgery for 12/17 cases, which resulted in histopathologic diagnoses. Follow-up ranged from 0 to 872 days postoperatively with a mean of 200 days with only 1 case having no follow-up. Eleven out of 17 cases (64.7%) had no documented recurrence following surgical excision. The overall complication rate was 35.3% (6/17 cases) and included orofacial/oroantral fistula formation, sinus flap suture reaction, and sinus flap mycosis.

**Discussion:**

Diagnosis and treatment of equine cystic masses of the maxilla, mandible, and/or incisive bones were greatly aided by assimilating oral exam and diagnostic imaging findings with histopathologic results.

## Introduction

Equine maxillofacial cystic and pseudocystic non-neoplastic lesions are relatively uncommon pathologies that can result in severe morbidity and, in some cases, mortality for the patient. Cystic and pseudocystic masses have been observed clinically with a diverse presentation in maxillomandibular location and patient signalment; previous publications are primarily confined to case reports and small series (1–6). Most lesions have previously been documented as unicameral or aneurysmal bone cysts that most commonly present in the mandible of young horses (7). Bone cysts are defined as benign intraosseous cavitated expansions that occur as a fluid filled mass surrounded by bone (7). This cavitated space is reported to contain either hemorrhagic or serosanguinous fluid surrounded by organizing fibrovascular tissue and reactive bone (7). The 2018 *World Health Organization Classification of Head and Neck Tumours* recognizes two types of bone cysts in humans “aneurysmal bone cyst” (ABC) and “simple bone cyst” (SBC) (8). Aneurysmal bone cysts have been most frequently reported in horses in the veterinary literature with one case report of a maxillary aneurysmal bone cyst in the maxilla of a young dog (1–4, 9, 32).

Dentigerous and radicular cysts can occur in any location associated with the teeth. Dentigerous cysts are defined as a cystic dilation of the dental follicle around an unerupted tooth due to fluid accumulation between the reduced enamel epithelium and the tooth crown in human and veterinary literature (10, 11). Dentigerous cysts are strongly suspected when an unerupted tooth is associated with a well-defined multilocular to unilocular radiolucent area with a sclerotic border on radiography (7). Diagnosis is confirmed via biopsy with histological confirmation of an epithelial lining, ± cytology of fluid with identification of epithelial cells, in combination with radiographic findings and identification of the presence of an unerupted tooth (7). Radicular cysts are defined as inflammatory odontogenic cysts associated with the reserve crown or root of a non-vital tooth that form secondary to chronic inflammation, and are the most common type of odontogenic cysts in humans (7, 10). Radicular cysts have been infrequently reported in the veterinary literature (12–15). Diagnosis of radicular cysts is suspected when oral exam and radiographs reveal the presence of a non-vital, endodontically or periodontally compromised tooth associated with an expansile fluid-filled lesion in humans and animals (7, 10, 11). Diagnosis is confirmed with histopathology of the cystic lining to differentiate these lesions from a periapical granuloma or periapical abscess (7).

While sinus cysts are the second most common cause of secondary sinusitis in the horse, there has been little documentation of other non-neoplastic cystic masses such as radicular, dentigerous, and bone cysts within the paranasal sinuses (16). Most reports of “dentigerous cysts” in the horse are actually periauricular temporal teratoma, which is a developmental disorder resulting from the aberrant migration of neural crest-derived ectomesenchymal stem cells from the first branchial arch (17, 18). This aberrant migration results in formation of dental tissue that may be enclosed by a cystic structure in the parietotemporal region. Temporal teratomas should be considered a separate lesion to dentigerous cysts because they originate outside of the normal dental arcade (7). When the literature was reviewed, true dentigerous cysts were reported to be associated with unerupted wolf teeth (19) and the paranasal sinuses (20, 21) or as a congenital odontogenic keratocyst (22). A mandibular radicular cyst has been reported in a mare (14). Overall, equine literature describing cystic masses of the incisive, maxillary and mandibular bones is sparse and often lacks sufficient detail for the reader to understand diagnostic features of these entities. The aim of this study was to retrospectively describe the clinical signs, imaging findings, histopathologic diagnoses, treatments, and clinical outcomes for cystic masses in equine incisive, maxillary, and mandibular bones.

## Materials and methods

### Case selection

Medical records from the Specialty Oral Pathology for Animals (SOPA) and Colorado State University Equine Dentistry and Oral Surgery Service were searched for incisive, maxillary, and mandibular equine cystic lesions. In addition, a questionnaire was sent to all equine diplomates of the American Veterinary Dental College seeking cases of equine cavitated/cystic lesions within the maxillofacial region. Inclusion criteria for the study were: cystic/cavitated lesions within the incisive, maxillary, and/or mandibular bones, skull radiographs and/or computed tomography (CT) imaging of the lesion, and complete medical records with patient history, description of the gross appearance of the lesion, histopathology report, treatments performed, and clinical outcomes. Exclusion criteria included masses that originated from the ethmoid and sphenopalatine sinuses, neoplastic lesions, and lesions that appeared to be cystic on diagnostic imaging but were found to be solid tissue lacking a cavity or cyst lumen during surgery. Primary sinus cysts and teratomas were excluded from the study.

The following data were recorded for each patient: breed, sex, age, location of the lesion, final diagnosis, gross description of the lesion, photographic documentation, clinical signs, relative lesion growth rate, case history, imaging descriptions (skull radiograph and CT), biopsy history, histopathology results, treatments performed, peri-operative medications, clinical outcomes, complications, and recheck information. Lesion locations were categorized as rostral maxilla (region containing all incisors and the second and third premolar teeth), caudal maxilla/paranasal sinus system (region containing the fourth premolar to the third molar teeth and all sinuses), rostral mandible (region containing all incisors and the incisor-premolar diastema), and body of the mandible (region containing all premolar and molar teeth). All diagnostic images were reviewed by a diplomate of the American Veterinary Dental College Equine Specialty (JR) and an equine dentistry and oral surgery resident (JK). The images were evaluated in communal, non-blinded fashion for presence or absence of tooth involvement, dysplastic teeth, unerupted teeth, irregular dental radiopacity, multiloculated vs. uniloculated appearance, well-circumscribed vs. poorly circumscribed margins, rarefication/cortical thinning of the surrounding bone, increased density/osteosclerosis of the adjacent bone vs. decreased density of the adjacent bone, sunburst reaction of the adjacent bone, osteoproductive appearance, increased soft tissue opacity, evidence of secondary sinusitis (fluid lines, increased sinus soft tissue opacity), and nasal passage involvement.

Histopathological evaluation was performed on tissues fixed in buffered formalin and decalcified with hydrochloric acid solution as needed. Tissues were processed routinely, embedded in paraffin, and 5 um sections were stained with hematoxylin and eosin. Original histopathologic evaluation was performed by a variety of diagnostic laboratories. All histopathologic reports were reviewed by a single board-certified pathologist (CB). Histopathologic reports were evaluated for the histopathologic diagnosis, presence or absence of epithelium, characterization of inflammation, and microscopic descriptions of the lesion, epithelium, bone, and dental material. Lesions were classified when possible to existing definitions by evaluating all clinical, radiological and histopathological findings. Lesion classification included dentigerous cyst (DTC), radicular cyst (RC), bone cyst (BC), and unclassified inflammatory lesion (UIL). Descriptive statistics were used to describe the data collected.

## Results

### Case population

Seventeen equine patient medical records from 6 institutions over a 5-year period (2017–2022) met the inclusion criteria with 16 patients having a singular lesion and 1 patient having multifocal maxillary and mandibular cystic lesions. The age range was 0.75–36 years with a median age of 8 years. The most common breed was the Thoroughbred (6) followed by Paint horses (3), Warmbloods (2), Andalusian (1), Quarter Horse (1), Lusitano (1), Standardbred (1), Arabian/Cob cross (1), and a pony (1). Male castrated horses were the most common sex (10) followed by female (4) and male (3) intact horses.

#### Diagnoses

Diagnosis of lesion etiology and type was made by assimilating histopathology reports with clinical findings and imaging results. The following cysts were diagnosed: dentigerous cyst (6), bone cyst (6), radicular cyst (3), and unclassified inflammatory lesion (2).

### Location of lesions

The most common lesion location was the body of the mandible (8) followed by caudal maxilla/sinuses (5), rostral maxilla (4), and rostral mandible (2). One patient had multifocal lesions of the rostral maxilla, caudal maxilla/sinuses, and the body of the mandible (Table 1). Dentigerous cysts occurred throughout the jaws including 2 cases within the rostral maxilla, 3 cases within the mandibular body, and 1 case with multifocal involvement throughout the dental arcades. The majority of bone cysts occurred in the body of mandible (4) with the remaining 2 cases occurring in the caudal maxilla/sinuses. The majority of radicular cysts occurred in the caudal maxilla/sinuses (2) with the 1 remaining case occurring in the rostral maxilla. Unclassified inflammatory lesions occurred in the rostral mandible (2).

**Table 1:** Signalment, location, and diagnosis of lesions.

Case #	Breed	Age (years)	Sex	Diagnosis	Location	Teeth involved*	Dental abnormalities
1	Andalusian	6.5	MC	DTC	Rostral maxilla	103–106	Unerupted 104
2	Quarter Horse	7	MC	DTC	Rostral maxilla	103–106	Unerupted 104
3	TB	1.66	FI	BC	Caudal maxilla/sinus	109–110	Dysplastic 109
4	TB	0.75	MI	BC	Mandibular body	807	No evidence of endodontic or periodontal pathology
5	Lusitano	8	MC	RC	Rostral maxilla	106–107	Dysplastic 106
6	TB	25	MC	UIL	Rostral mandible	304–404	No evidence of endodontic or periodontal pathology
7	Standardbred	1.5	MC	BC	Caudal maxilla/sinus	608–210	Dysplastic 209
8	TB	24	FI	BC	Mandibular body	308–309	No evidence of endodontic or periodontal pathology
9	TB	3	MI	DTC	Mandibular body	306–307	Unerupted 306
10	Warmblood	18	MC	BC	Mandibular body	408–411	Complicated crown fractures 408, 409, 410
11	TB	16	MC	BC	Mandibular body	None	No dental involvement
12	Paint	36	MC	UIL	Rostral mandible	401–304	Missing 303 Complicated crown fracture 304
13	Paint	2.5	MC	DTC	Mandibular body	306–308	8 mm periodontal pocket 707/708 Unerupted 306, 307
14	Arabian/Cob	11	FI	RC	Caudal maxilla/sinus	208–211	210 periapical abscessation
15	Paint	20	MC	RC	Caudal maxilla/sinus	109–111	Complicated crown fracture 209
16	Warmblood	18	MC	DTC	Mandibular body	308	Unerupted 307
17	Pony	0.75	FI	DTC (3)	Rostral maxilla, Caudal maxilla/sinus, Mandibular body	All premolars and molars	Unerupted and malpositioned 109, dysplastic and abnormal orientation of abnormally developed cheek teeth

*Modified Triadan system of tooth numbering (31). TB, thoroughbred; MC, male castrated; MI, male intact; FI, female intact; DTC, dentigerous cyst; BC, bone cyst; RC, radicular cyst; UIL, unclassified inflammatory lesion.

### Clinical signs and oral exam findings

**Figure 1:**
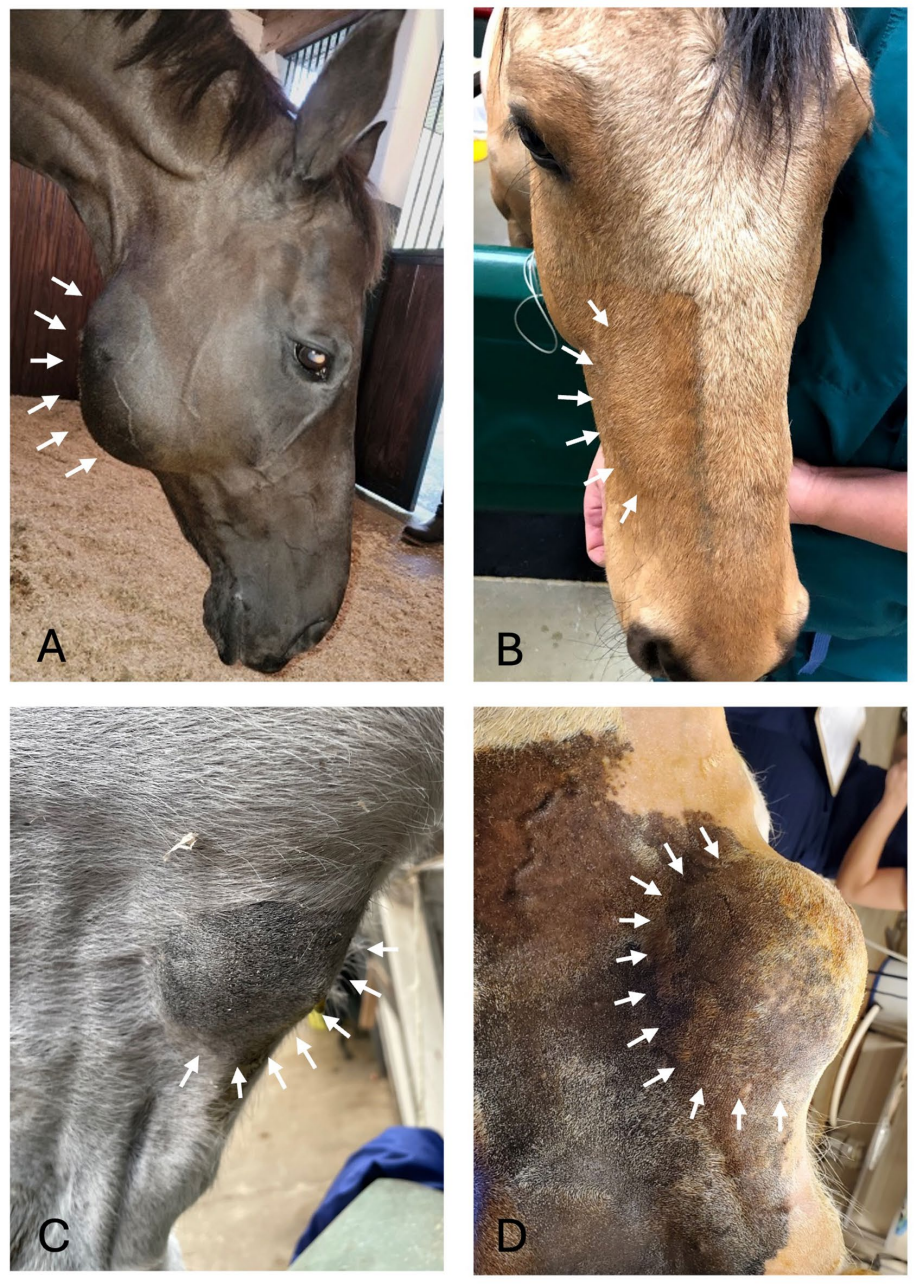
Facial swelling. **(A)** Case 11 right caudal mandibular facial swelling (white arrows) secondary to bone cyst (image courtesy of Easley equine dentistry). **(B)** Case 5 right rostral maxillary facial swelling (white arrows) secondary to radicular cyst. **(C)** Case 8 left mandibular facial swelling (white arrows) secondary to bone cyst. **(D)** Case 16 left mandibular facial swelling (white arrows) secondary to dentigerous cyst.

Oral examination findings in the region of the lesion are listed in Table 1. The clinical signs of all cases are listed in Tables 2, 3 according to mass type and location. The most common clinical sign was facial swelling which accounted for 13/17 (76.5%) cases (Figure 1). Missing or unerupted teeth was the second most common clinical sign affecting 7/17 (41.2%) cases. Nasal discharge and respiratory issues (stertor, increased respiratory rate or effort) were the third most common clinical signs and affected 4/17 (23.5%) cases. Other clinical signs included halitosis (3), cystic cavity packed with debris (2), and orofacial draining tracts (1). All dentigerous cysts had facial swelling and unerupted teeth (6/6). Bone cysts were accompanied by facial swelling (4/6, 66.7%) and respiratory issues (2/6, 33.3%). Complicated crown fractures were present in 3/17 (17.6%) cases and were diagnosed in a single case of UIL, BC and RC. A periapical abscess was diagnosed in a single case of a RC. Dysplastic teeth were diagnosed in 4/17 (23.5%) cases. There was no evidence of endodontic or periodontal pathology in 4/17 (23.5%) cases diagnosed in 3 BC and 1 UIL.

**Table 2:** Clinical signs according to lesion type.

Type of mass	Facial swelling	Nasal discharge	Respiratory issues	Orofacial draining tract	Halitosis	Cystic cavity packed with debris	Missing/unerupted teeth
Dentigerous cyst*	6 /6 (100%)	1/6 (16.7%)	1/6 (16.7%)	1/6 (16.7%)	0/6 (0%)	0/6 (0%)	6/6 (100%)
Radicular cyst	2/3 (66.7%)	2/3 (66.7%)	1/3 (0%)	0/3 (0%)	1/3 (33.3%)	0/3 (0%)	0/3 (0%)
Bone cyst	4/6 (66.6%)	1/6 (16.7%)	2/6 (33.3%)	0/6 (0%)	0/6 (0%)	0/6 (0%)	0/6 (0%)
Unclassified inflammatory lesion	1/2 (50%)	0/2 (0%)	0/2 (0%)	0/2 (0%)	2/2 (100%)	2/2 (100%)	1/2 (50%)
Total	13/17 (76.5%)	4/17 (23.5%)	4/17 (23.5%)	1/17 (5.9%)	3/17 (11.8%)	2/17 (11.8%)	7/17 (41.2%)

*Includes multifocal maxillary and mandibular dentigerous cysts case #17.

**Table 3:** Clinical signs according to lesion location.

Location of mass	Facial swelling	Nasal discharge	Respiratory issues	Orofacial draining tract	Halitosis	Cystic cavity packed with debris	Missing/unerupted teeth
Body of mandible	7/8 (87.5%)	0/8 (0%)	0/8 (0%)	1/8 (12.5%)	0/8 (0%)	0/8 (0%)	3/8 (12.5%)
Caudal maxilla/sinuses	3/5 (60%)	4/5 (80%)	4/5 (80%)	0/5 (0%)	1/5 (20%)	0/5 (0%)	1/5 (20%)
Rostral maxilla	4/4 (100%)	1/4 (25%)	1/4 (25%)	0/4 (0%)	0/4 (0%)	0/4 (0%)	3/4 (75%)
Rostral mandible	1/2 (50%)	0/2 (0%)	0/2 (0%)	0/2 (0%)	2/2 (100%)	2/2 (100%)	1/2 (50%)
Total*	15/19 (78.9%)	5/19 (26.3%)	5/19 (26.3%)	1/19 (5.3%)	3/19 (15.8%)	2/19 (10.5%)	8/19 (42.1%)

*Case 17 includes maxillary and mandibular masses increasing the location of mass count to 19.

### Radiographic findings

**Figure 2:**
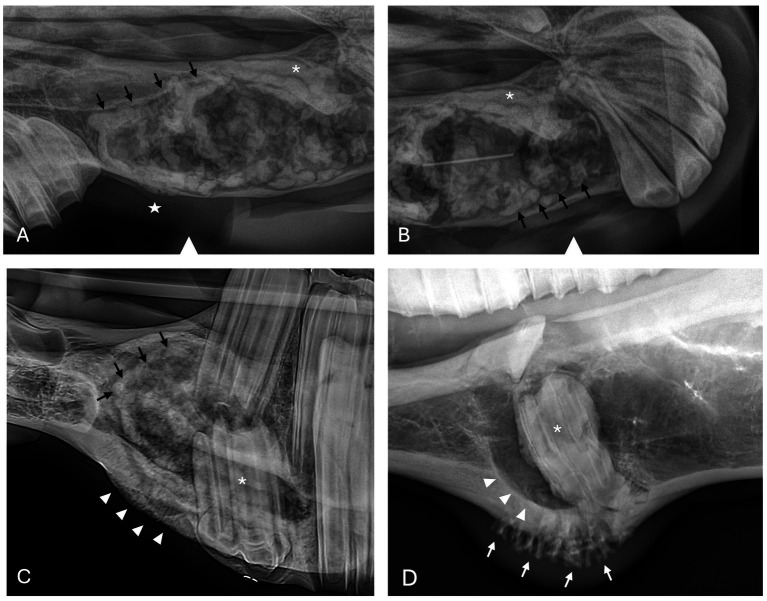
Dentigerous cyst radiographic findings. **(A,B)** Case 2 multiloculated, well circumscribed dentigerous cyst containing irregular dental material (black arrows) associated with unerupted and dysplastic 104 (white asterisk), with rarefication of surrounding bone (white star) Images courtesy of Dr. Robert Baratt. **(C)** Case 9 multiloculated, well circumscribed dentigerous cyst containing irregular dental material (black arrows) associated with unerupted and dysplastic 306 (white asterisk) with osteosclerosis of adjacent bone (white arrowheads) Image courtesy of Easley equine dentistry. **(D)** Case 16 unilocular, well circumscribed dentigerous cyst associated with unerupted and dysplastic 307 (white asterisk), with osteosclerosis of adjacent bone (white arrowheads), and sunburst reaction (white arrows).

**Figure 3:**
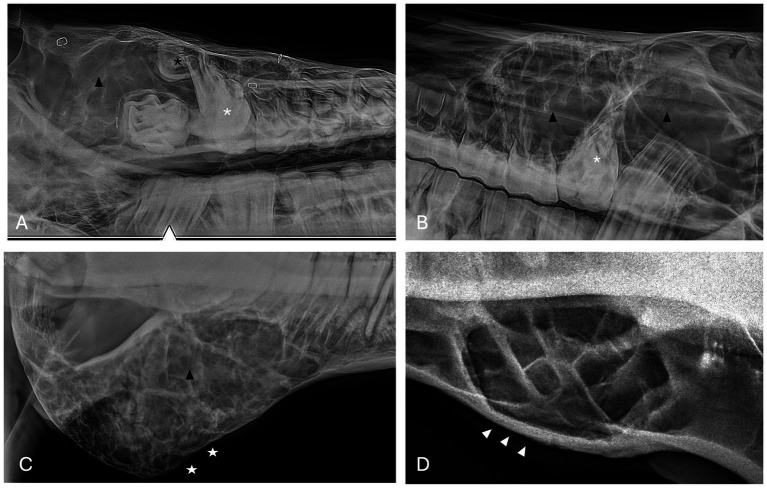
Bone cyst radiographic findings. **(A)** Case 3 multiloculated, poorly circumscribed bone cyst associated with dysplastic right maxillary first molar (white asterisk) and denticle (black asterisk) with increased soft tissue opacity (black arrowhead) and secondary sinusitis. Image courtesy of Easley equine dentistry. **(B)** Case 7 multiloculated, well circumscribed left caudal maxillary/paranasal sinus bone cyst associated with dysplastic left maxillary first molar (white asterisk) with increased soft tissue opacity (black arrowheads) and secondary sinusitis. Image courtesy of Easley equine dentistry. **(C)** Case 10 right caudal mandibular multiloculated, well circumscribed right caudal mandibular bone cyst with increased soft tissue opacity (black arrowhead) and rarefication of surrounding bone (white stars). Image courtesy of Easley equine dentistry. **(D)** Case 8 multiloculated, well circumscribed left mandibular bone cyst with osteosclerosis of surrounding bone (white arrowheads).

**Figure 4:**
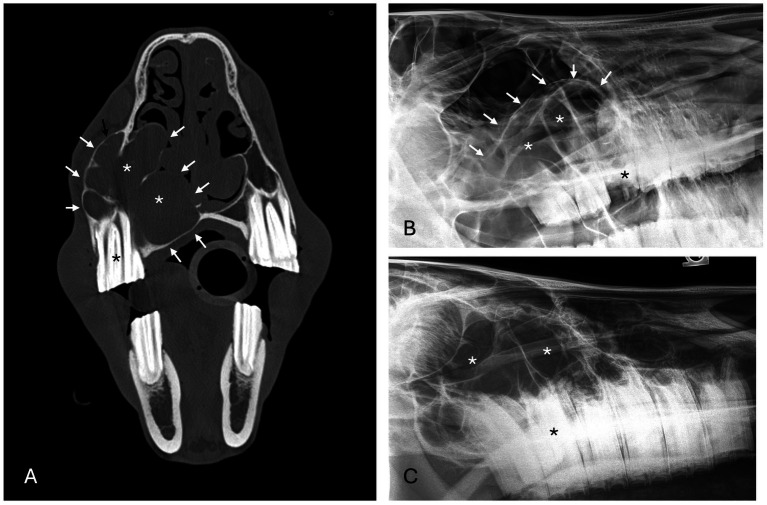
Radicular cyst radiographic findings. **(A)** Case 5 multiloculated, well circumscribed (white arrows) radicular cyst associated with dysplastic right maxillary second premolar (black asterisk) with increased soft tissue opacity (white asterisks), nasal passage involvement, and rarefication of surrounding bone (black arrow). **(B)** Case 15 multiloculated, well circumscribed (white arrows) radicular cyst associated with complicated crown fracture of left maxillary first molar (black asterisk) with associated increased soft tissue opacity (white asterisks). **(C)** Case 14 multiloculated, poorly circumscribed radicular cyst associated with a periapical lucency of the left maxillary second molar (black asterisk) with associated increased soft tissue opacity (white asterisks).

Radiographic findings are reported based on lesion type (Table 4) and location (Table 5). The most prevalent radiographic finding was tooth involvement in 16/17 (94.1%) cases followed by multiloculated lesions and increased soft tissue opacity in 14/17 (82.4%) cases, respectively (Figures 2–4). Rarefication of the surrounding bone (cortical thinning) was the third most common imaging finding identified in 13/17 (76.5%) of cases. All lesions had tooth involvement except for 1 bone cyst lesion. Radiographically unerupted teeth were 100% associated with dentigerous cysts (6/6) of which 83% (5/6) of the unerupted teeth were dysplastic (Figure 2). Thirty-three percent (2/6) of dentigerous cysts were associated with additional irregular dental material, and both of these lesions were located within the rostral maxilla (Figure 2). Dysplastic teeth also occurred in 2/6 (33%) of bone cysts (Figure 3). Abnormal dental findings were more closely linked to lesion type than location in the mouth.

**Table 4:** Radiographic findings based on lesion type.

Radiographic findings	Dentigerous cyst	Radicular cyst	Bone cyst	UIL	Total
Tooth involvement	6/6 (100%)	3/3 (100%)	5/6 (83.3%)	2/2 (100%)	16/17 (94.1%)
Dysplastic teeth	5/6 (83.3%)	0/3 (0%)	2/6 (33.3%)	0/2 (0%)	7/17 (41.2%)
Unerupted teeth	6/6 (100%)	0/3 (0%)	0/6 (0%)	0/2 (0%)	6/17 (35.3%)
Irregular dental radiopacity	2/6 (33.3%)	0/3 (0%)	0/6 (0%)	0/2 (0%)	2/17 (11.8%)
Multiloculated	5/6 (83.3%)	3/3 (100%)	6/6 (100%)	0/2 (0%)	14/17 (82.4%)
Unilocular	1/6 (16.6%)	0/3 (0%)	0/6 (0%)	2/2 (100%)	3/17 (17.6%)
Well-circumscribed	5/6 (83.3%)	2/3 (66.6%)	5/6 (83.3%)	2/2 (100%)	14/17 (82.4%)
Poorly circumscribed	1/6 (16.6%)	1/3 (33.3%)	1/6 (16.6%)	0/2 (0%)	3/17 (17.6%)
Rarefication of surrounding bone	5/6 (83.3%)	1/3 (33.3%)	5/6 (83.5%)	2/2 (100%)	13/17 (76.5%)
Osteosclerosis of adjacent bone	3/6 (50%)	1/3 (33.3%)	2/6 (33.3%)	1/2 (50%)	7/17 (41.2%)
Osteolysis of adjacent bone	1/6 (16.6%)	0/3 (0%)	4/6 (66.6%)	1/2 (50%)	6/17 (35.3%)
Osteoproductive	4/6 (66.6%)	0/3 (0%)	0/6 (0%)	0/2 (0%)	4/17 (23.5%)
Sunburst reaction	1/6 (16.6%)	0/3 (0%)	0/6 (0%)	0/2 (0%)	1/17 (14.3%)
Increased soft tissue opacity	5/6 (83.3%)	3/3 (100%)	4/6 (66.6%)	2/2 (100%)	14/17 (82.4%)
Secondary sinusitis	1/6 (16.6%)	2/3 (66.6%)	2/6 (33.3%)	0/2 (0%)	5/17 (29.4%)
Nasal passage involvement	1/6 (16.6%)	2/3 (66.6%)	2/6 (33.3%)	0/2 (0%)	5/17 (29.4%)

Unclassified inflammatory lesion (UIL).

**Table 5:** Radiographic findings based on lesion location.

Radiographic findings	Body of mandible	Caudal maxilla/sinus	Rostral maxilla	Rostral mandible	Total
Tooth involvement	7/8 (87.5%)	5/5 (100%)	4/4 (100%)	2/2 (100%)	18/19 (94.7%)
Dysplastic teeth	4/8 (50%)	3/5 (60%)	2/4 (50%)	0/2 (0%)	9/19 (47.4%)
Unerupted teeth	4/8 (50%)	1/5 (16.7%)	3/4 (75%)	0/2 (0%)	8/19 (42.1%)
Irregular dental radiopacity	0/8 (0%)	0/5 (0%)	2/4 (50%)	0/2 (0%)	2/19 (10.5%)
Multiloculated	7/8 (87.5%)	5/5 (100%)	4/4 (100%)	0/2 (0%)	16/19 (84.2%)
Unilocular	1/8 (12.5%)	0/5 (0%)	0/4 (0%)	2/2 (100%)	3/19 (15.8%)
Well-circumscribed	7/8 (87.5%)	3/5 (60%)	3/4 (75%)	0/2 (0%)	13/19 (68.4%)
Poorly circumscribed	1/8 (12.5%)	3/5 (60%)	1/4 (25%)	0/2 (0%)	5/19 (26.3%)
Rarefication of surrounding bone	6/8 (75%)	3/5 (60%)	4/4 (100%)	2/2 (100%)	15/19 (78.9%)
Osteosclerosis of adjacent bone	5/8 (62.5%)	0/5 (0%)	1/4 (25%)	1/2 (50%)	7/19 (36.8%)
Osteolysis of adjacent bone	3/8 (37.5%)	3/5 (60%)	1/4 (25%)	1/2 (50%)	8/19 (42.1%)
Osteoproductive	2/8 (25%)	0/5 (0%)	2/4 (50%)	0/2 (0%)	4/19 (21%)
Sunburst reaction	1/8 (12.5%)	0/5 (0%)	0/4 (0%)	0/2 (0%)	1/19 (5.3%)
Increased soft tissue opacity	4/8 (50%)	5/5 (100%)	4/4 (100%)	2/2 (100%)	15/19 (78.9%)
Secondary sinusitis	0/8 (0%)	5/5 (100%)	1/4 (25%)	0/2 (0%)	6/19 (31.6%)
Nasal passage involvement	0/8 (0%)	4/5 (80%)	2/4 (50%)	0/2 (0%)	6/19 (31.6%)

**Figure 5:**
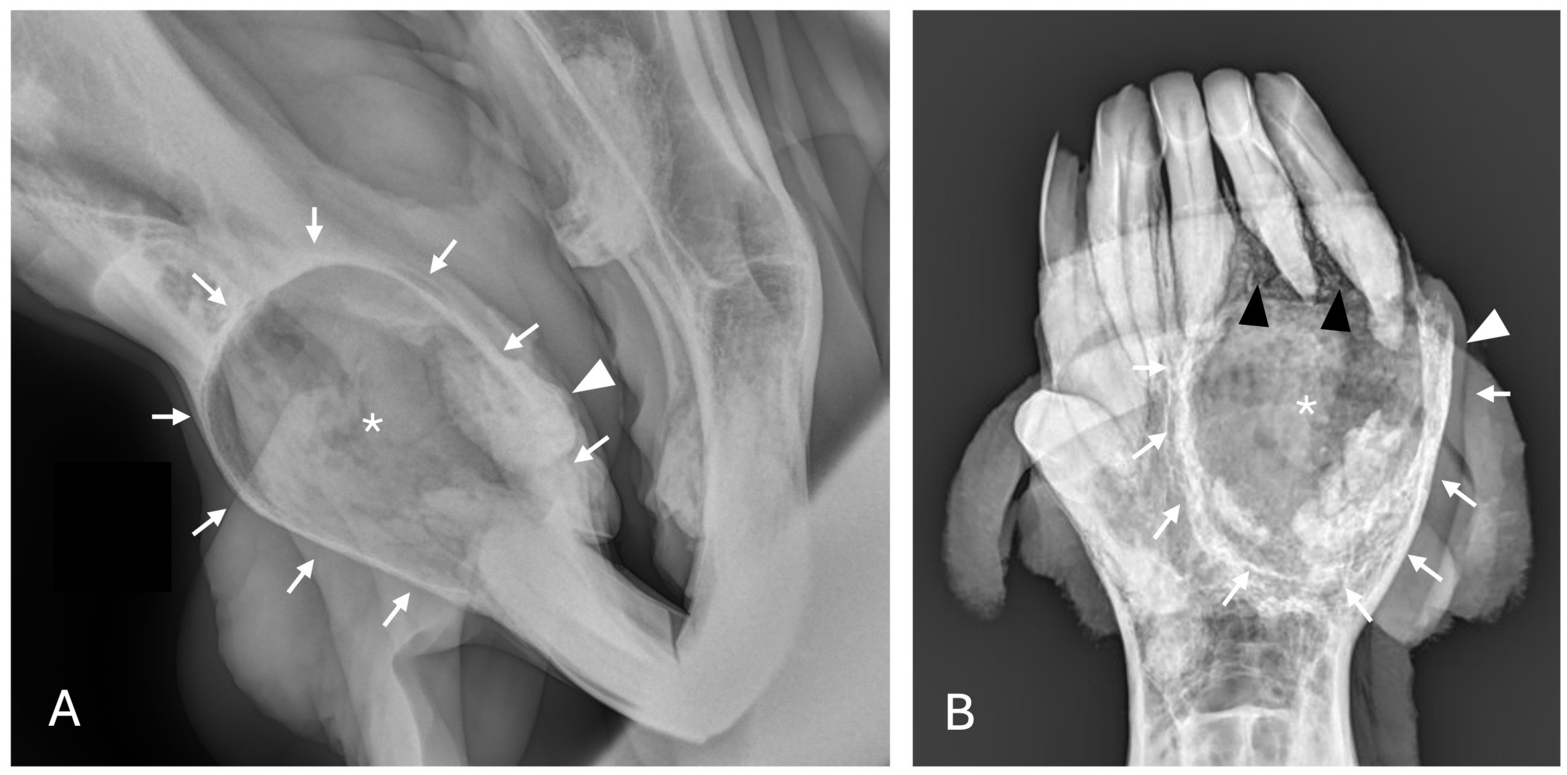
Unclassified, radiolucent inflammatory lesions **(A)** Case 6 unilocular, well circumscribed (white arrows) inflammatory lesion involving the mandibular incisors and canines with associated increased soft tissue opacity (white asterisk), osteosclerosis of adjacent bone and cystic cavity packed with debris. Image courtesy of Dr. Laurelyn Kenner. **(B)** Case 12 unilocular, well circumscribed (white arrows), inflammatory lesion involving left mandibular incisors and canine with increased soft tissue opacity (white asterisk), osteosclerosis of adjacent bone (white arrowhead) and osteolysis (black arrowheads). Image courtesy of Dr. Jessica Hunt.

Rarefication of the surrounding bone was the most common radiographic bony change identified in 13/17 (76.5%) of cases (Figure 2). Osteosclerosis of adjacent bone occurred in 7/17 (41.2%) of cases while osteolysis of adjacent bone occurred in 6/17 (35.3%) of cases (Figure 5). Rarefication of the surrounding bone was most commonly associated with dentigerous cysts (5/6; 83.3%) and bone cysts (5/6; 83.3%). Osteosclerosis of adjacent bone was observed most frequently in the body of the mandible with 5/7 (71.4%) of cases affected. Osteolysis of adjacent bone was most frequently observed in the caudal maxilla/paranasal sinuses (3/5; 60%) and in bone cysts (4/6; 66.6%). Dentigerous cysts were the only type of lesion in this study to have an osteoproductive appearance with 4/6 (66.6%) cases of dentigerous cysts affected. Sunburst reaction was observed in a single case of a mandibular body dentigerous cyst (Figure 2).

Increased soft tissue opacity was most commonly observed in dentigerous cysts (5/6) and unclassified, radiolucent inflammatory lesions (2/2) (Figure 5). All lesions located in caudal maxilla/paranasal sinuses, rostral maxilla and rostral mandible had increased soft tissue opacity (Figures 3, 4). Nasal passage involvement was observed in 4/5 cases in the caudal maxilla/paranasal sinuses and in 1/3 of cases located in the rostral maxilla (Figure 4). Radicular cysts were the most common lesion type associated with nasal passage involvement with 2/3 cases affected (Figure 4). Secondary sinusitis was observed in all cases located in the caudal maxilla/paranasal sinuses. Sixty-six percent of radicular cysts (2/3) demonstrated secondary sinusitis.

### Histopathologic findings

A lesion was diagnosed as a cyst when epithelium was present along the luminal surface of the lesion; however, final classification of the cyst type relied heavily on correlation with clinical and diagnostic imaging findings. If a cyst was associated with an unerupted tooth, it was diagnosed as a dentigerous cyst. There were 6 dentigerous cysts identified in the study. Detailed epithelial descriptions were present in 4/6 dentigerous cyst samples. Epithelium was described as stratified squamous epithelium without keratinization in 2 cases, odontogenic epithelium in 1 case, and respiratory epithelium with variable squamous metaplasia and focal ulceration in 1 case. Dentigerous cystic lesions were overall described as having a loose fibrovascular stroma with multifocal regions of hemorrhage and hemosiderin deposits surrounding a cavitated space. When bone descriptions were included in the dentigerous cyst reports (5/6), it was described as remodeled with characteristics ranging from dense bone with areas of mature lamellar organization with smoothly contoured edges to reactive woven bone. Mineralized tissue was evaluated and determined to not represent denticles, thereby excluding odontoma. Finally, inflammation was associated with dentigerous cysts, and it was characterized as mild with a mixed population of leukocytes in 5/6 cases with 1 case demonstrating robust inflammatory infiltrates.

**Figure 6:**
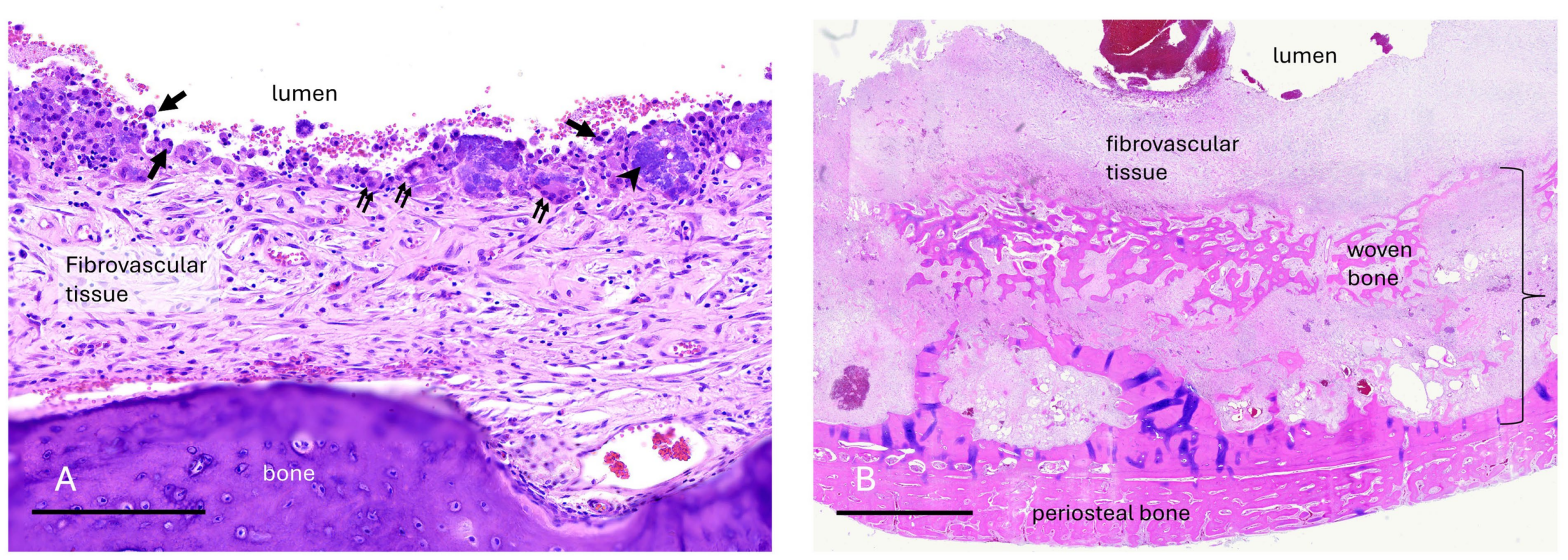
Case 7 **(A)** Maxillary bone cyst from a young horse. A high magnification image of the luminal surface lacks an epithelial lining. Instead, fibrovascular tissue that faces the lumen has an irregular layer of macrophages (arrows), multinucleate osteoclast-like giant cells (double arrows), granular basophilic “chondroid-like” matrix (arrowhead), and erythrocytes from hemorrhage. Bar – 200um, HE stain. Case 10 **(B)** Mandibular bone cyst from an adult horse. A low magnification image includes the entire thickness of the lesion wall including a thin, compacted layer of reactive periosteal bone. Original cortical bone (approximated by the bracket) is thickened and replaced by reactive woven bone and fibrovascular connective tissue. The lumen contains hemorrhage. There is neither epithelium nor an organized cellular/debris layer along the luminal edge. Bar – 3 mm, HE stain.

Histopathology supported the diagnosis for 6 bone cysts. The bone cyst lesions were histologically characterized by the presence of a fibrovascular stroma with embedded plates or thin trabeculae of well-organized or porous woven bone. All bone cyst cases had evidence of a pseudocystic lining that was defined as fibrovascular tissue of the luminal surface that contained macrophages and multi-nucleate giant cells and/or amorphous mineral deposits (Figure 6). Macrophages and multi-nucleate giant cells were present in 3/6 cases and 2/6 cases demonstrated macrophages with no multinucleate giant cells. Amorphous mineral deposits were present within the pseudocyst lining in 3/6 cases. Except for macrophages and multinucleate giant cells, no other inflammatory cells were present in the pseudocyst lining for 4/6 cases while mild lymphoplasmacytic inflammation was present in 2/6 cases.

**Figure 7:**
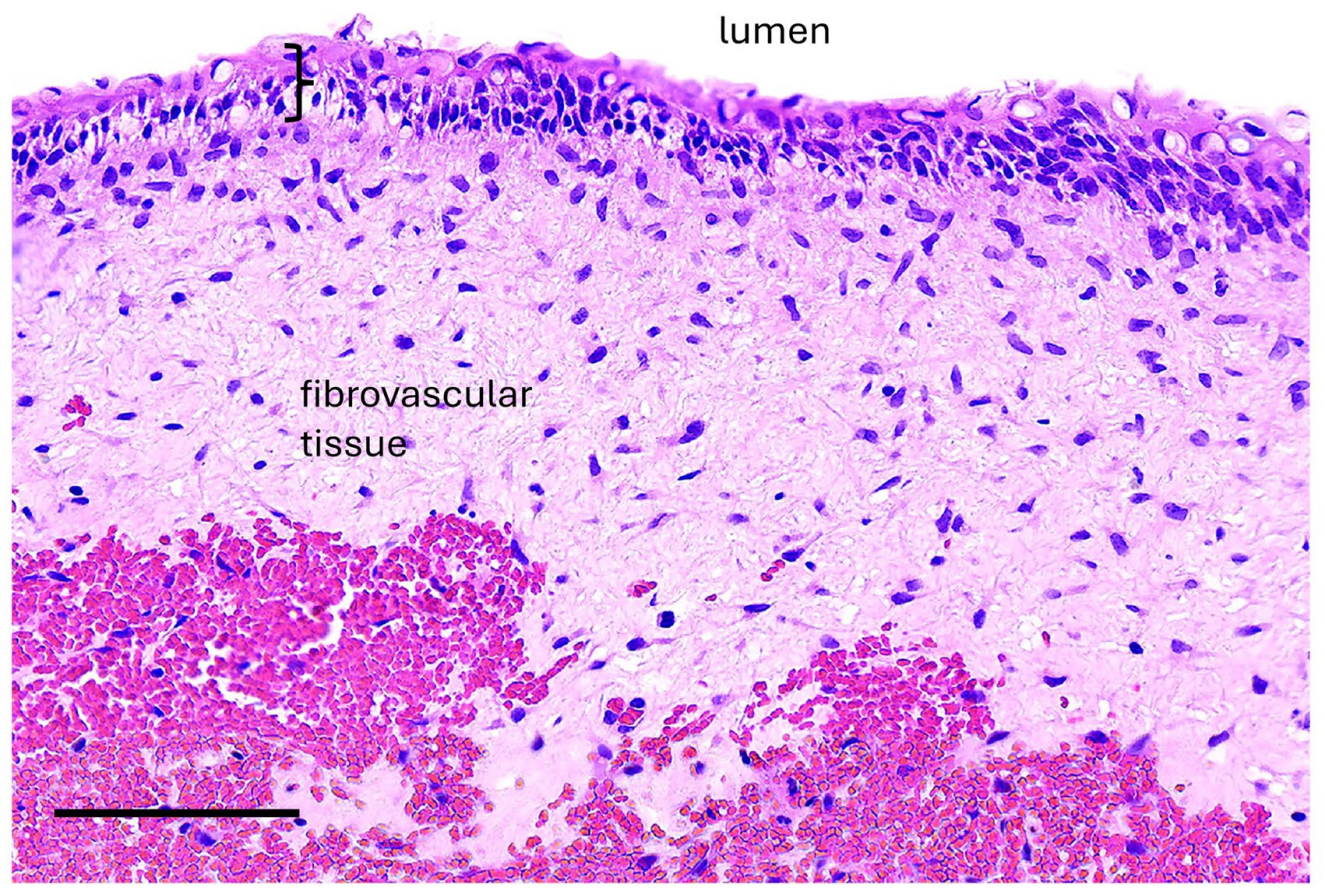
Case 5, Maxillary radicular cyst. A high magnification image of the cyst lining has epithelium (bracket) supported by fibrovascular connective tissue with hemorrhage (hemorrhage is likely an artifact of surgical enucleation). The epithelium is transitional between non-keratinized stratified squamous and pseudostratified, although cilia are not evident. Bar – 100 um, HE stain.

If a cyst was associated with an erupted dysplastic and/or diseased tooth causing paradental inflammation, a radicular cyst was diagnosed. Radicular cysts were diagnosed in 3 cases. Two maxillary lesions had respiratory epithelium with one case demonstrating squamous metaplasia. The other rostral maxillary radicular cyst had well-differentiated stratified squamous epithelium with no keratinization although low cuboidal epithelial cells were noted in the biopsy sample from a third procedure after recurrence and persistence of the cyst (Figure 7). Mixed lymphoplasmacytic and neutrophilic inflammation was present to varying degrees in all samples.

**Figure 8:**
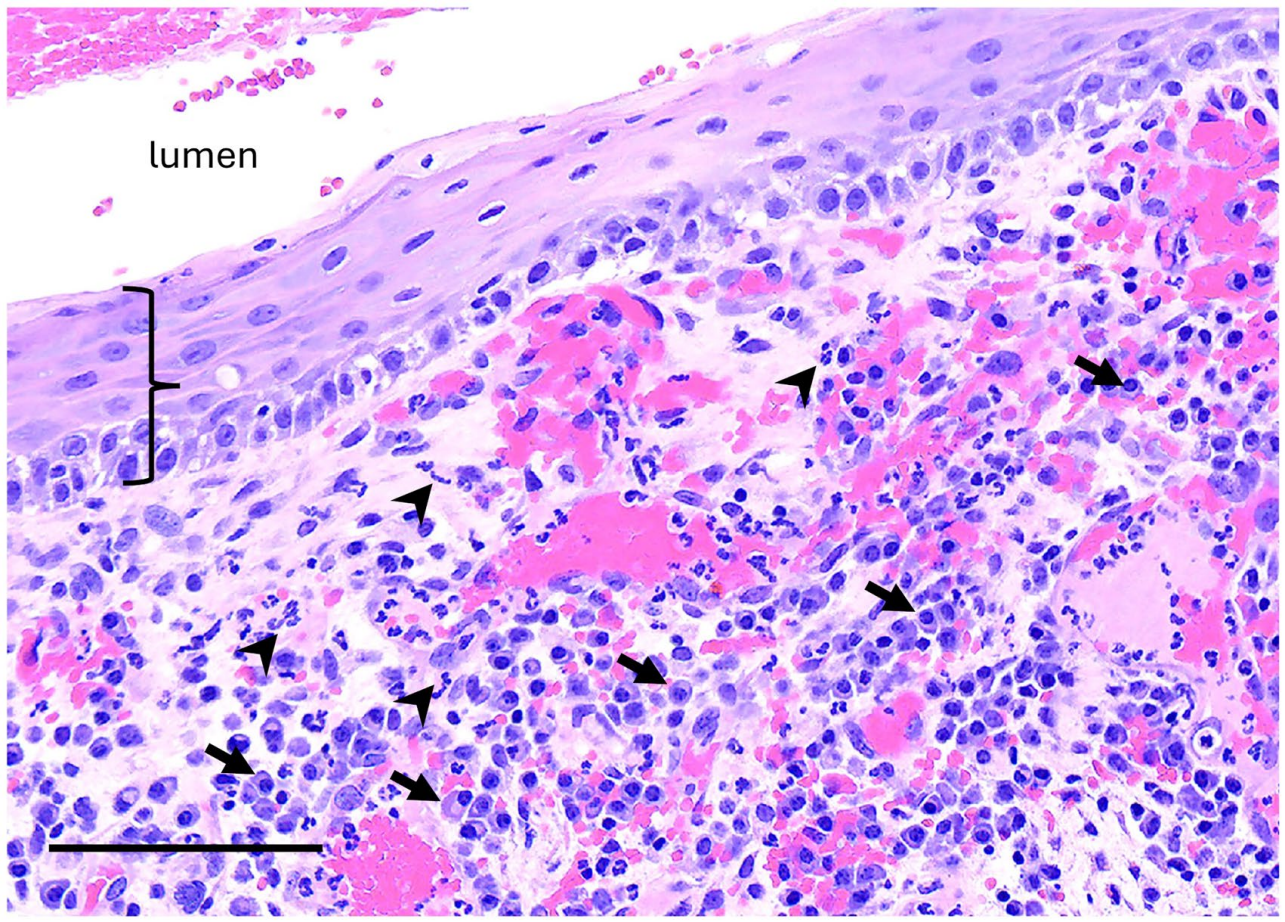
Unclassified radiolucent inflammatory lesion from the rostral mandible. A high magnification image of the luminal surface has non-keratinized stratified squamous epithelium (bracket) supported by fibrovascular connective tissue with robust inflammation including many plasma cells (arrows) and neutrophils (arrowheads). Bar – 100um, HE stain.

No definitive histologic diagnosis was able to be made for both cases of unclassified inflammatory lesions. Case 6 was reported as inflammatory epithelialized periapical abscess or abscessed cyst that had tissue lining the bony cavity described as granulation tissue and fibrosis with chronic inflammation and partial epithelial lining. The epithelium was characterized as nonkeratinized stratified squamous epithelium (Figure 8). Case 12 was reported as an inflammatory pseudocyst with secondary edema, fibrosis and mild bone remodeling. No epithelial lining was present in the sample. The inflammation of both lesions was characterized as lymphoplasmacytic with neutrophilic infiltrates and macrophages.

### Treatment and clinical outcome

Incisional biopsies were initially performed in 5/17 cases while the remainder of the cases had excisional biopsies performed at the time of definitive surgery (See Table 6). The histopathology reports for three of the incisional biopsy cases were consistent with a cystic lesion, but the pathologist was unable to provide a diagnostic interpretation for 1 dentigerous cyst and 1 bone cyst. Out of the 5 cases with incisional biopsies, one case had an incisional biopsy performed with a Galt trephine, which resulted in a definitive diagnosis for 1 bone cyst. A core incisional biopsy in another case showed only inflammation for 1 bone cyst, and a radicular cyst case had an unsuccessful incisional biopsy attempt under general anesthesia. Excisional biopsy samples obtained at the time of definitive surgery resulted in clear histopathologic descriptions of the lesions in all cases.

**Table 6:** Biopsy type, treatment, follow up and lesion recurrence.

Case number mass type	Biopsy type	Treatment	Time to first recheck (days)	Recurrence	Surgical and postoperative complications	Additional procedure(s)	Total follow up (days)
1: DTC	Excisional	Aggressive debridement, extraction	38	Yes	None	Aggressive debridement	70
2: DTC	Incisional	Aggressive debridement, extraction	180	No	None	None	180
3: BC	Excisional	Maxillary and nasofrontal sinus flaps, aggressive debridement	81	No	None	None	81
4: BC	Excisional	Aggressive debridement	0	Unknown	Unknown	None	0
5: RC	Incisional Excisional	Aggressive debridement	83	Yes*	Temporary facial nerve paresis	2nd: Extraction 3rd: Aggressive debridement and RNH removal	872
6: UIL	Excisional	Aggressive debridement	39	No	None	None	39
7: BC	Excisional	Aggressive debridement	47	No	None	None	47
8: BC	Incisional Excisional	Aggressive debridement	208	No	None	None	326
9: DTC	Excisional	Aggressive debridement, extraction	36	Yes	None	Not recorded	36
10: BC	Incisional Excisional	Aggressive debridement	38	No	None	None	320
11: BC	Incisional Excisional	Aggressive debridement	13	Unknown	Mandibular ramus stress fracture secondary to mass excision	None	13
12: UIL	Excisional	Rostral mandibulectomy	30	No	None	None	365**
13: DTC	Excisional	Aggressive debridement, extraction	36	No	Multiple sequestra and OFF	Repeated debridement of sequestra and OFF	248
14: RC	Excisional	Nasofrontal sinus flap, aggressive debridement, extraction	33	No	Suture osteitis Secondary fungal sinusitis	Sinus flap suture reaction debridement, sinus fungal plaque debridement	66
15: RC	Excisional	Rostral maxillary and nasofrontal sinus trephinations, aggressive debridement	216	No	None	None	216
16: DTC	Excisional	Aggressive debridement, extraction	40	No	Actinomyces osteomyelitis OFF	repeated OFF debridement Surgical intraoral fistula repair	178
17: DTC	Excisional	Maxillary sinus flap, aggressive debridement, extraction	181	Yes	Bilateral oroantral fistulas	Maxillary sinus flap, extraction, oroantral fistula management	343***

*Recurrence following second procedure. **Euthanized due to unrelated issues. ***Euthanized due to associated pathology. DTC, dentigerous cyst; BC, bone cyst; RC, radicular cyst; UIL, unclassified inflammatory lesion; RNH, reactive nodular hypercementosis; T/U, unerupted tooth; OFF, orofacial fistula.

Definitive treatment in 16/17 cases constituted aggressive surgical debridement of cystic structures and lining along with preservation of surrounding healthy cortical/cancellous bone. In the cases where unerupted teeth were present, surgical access to the tooth was acquired, and the unerupted tooth was extracted along with debridement of the cystic lining. In 1 case of an UIL, a rostral mandibulectomy was performed. This was the only case in which margins were attempted due to concern that a neoplastic process was present. For cases where a cystic lining was appreciated at the time of surgery, the lining was debrided as completely as possible and submitted for histopathology. When a cystic lining was not appreciated, the visible pathologic structures were debrided and submitted for histopathology.

Postoperative antimicrobial or analgesic management was not noted in the records for 6/17 cases. Of the 11 cases where postoperative medications were noted in the records, all 11 received postoperative antimicrobial and analgesic therapy. Antimicrobial drug selection varied greatly and included trimethoprim sulfa, ceftiofur, doxycycline, and metronidazole in varying combinations or as solo agents for an average duration of 14 days. Flunixin meglumine and phenylbutazone were the analgesic medications utilized ranging in duration from 3 to 5 days.

Follow up ranged from 0 to 872 days postoperatively with a median of 178 days. Only 1 case had no follow up. Mass recurrence was noted in 4/17 (23.5%) of cases following definitive surgical treatment (see Table 6). Three out of the four cases with recurrence were dentigerous cysts that recurred following extraction of the associated unerupted tooth and aggressive lesion debridement. One radicular cyst recurred following extraoral surgical debridement of the lesion and intraoral extraction of the associated tooth. One of the four cases with recurrence, case 5, demonstrated no further recurrence following two additional procedures with a maximum follow up of 872 days (radicular cyst). In case 1, there was no further follow up after a second procedure was performed at 70 days postoperatively from the initial procedure. Case 9 did not have any records of a second procedure being performed after recurrence was noted radiographically at the 36 day recheck. Case 17 was euthanized due to recurrence of her generalized dentigerous cysts and persistent oroantral fistulae 343 days following her initial diagnosis and treatment.

Intraoperative surgical complications and long-term sequelae were noted in 6/17 (35.3%) cases and included orofacial fistula, oroantral fistula, sinus flap suture reactions, temporary facial nerve paresis, sinus flap mycosis, and a single case of a mandibular ramus stress fracture secondary to mass excision (see Table 6). The 3 cases with persistent fistula formation were dentigerous cysts (2 mandibular and 1 maxillary) that formed following surgical extraoral extraction and lesion debridement. The mandibular persistent orofacial fistulae were able to be resolved with repeated debridement, sequestrum removal, and intraoral surgical repair via a pedicle mucoperiosteal flap (case 16). The case with oroantral fistula formation had multifocal persistent fistulae with feed packing into the sinuses that lead to the decision to humanely euthanize the horse (case 17). The sinus flap suture reaction and flap mycosis occurred in a horse with a radicular cyst (Case 14) that was successfully treated without recurrence. The case of a mandibular vertical ramus stress fracture occurred secondary to removal of a mandibular body bone cyst with an osteotome and mallet (Case 11). The fracture propagated along the caudodorsal aspect of the masseteric fossa adjacent to the mandibular cortex and extended to the mandibular condyle. The fracture was managed conservatively with no further complications reported but follow up was limited. Case 5 developed postoperative facial nerve paresis secondary to buccotomy and buccal alveolectomy which resolved within a few months. Finally, case 14 had a suture reaction managed with surgical debridement, antimicrobial, and anti-inflammatory therapy, while the fungal mycosis was treated with debridement of the fungal plaques via sinoscopy.

## Discussion

The goal of this study was to provide clinicians with clinically relevant information to aid in the diagnosis and management of equine cysts located in the region of the teeth. Three types of cystic or pseudocystic lesions were identified: DTC (35%), bone cyst (35%), and RC (18%). No definitive diagnosis was determined for two lesions, which were designated as UIL (12%). Diagnosis of cysts and pseudocysts requires correlation of histopathologic descriptions with diagnostic imaging and oral examination findings. Identification of a fractured tooth, pulp exposure, or other evidence of a non-vital tooth on oral exam was necessary to diagnose a radicular cyst. Dentigerous cysts were always associated with unerupted teeth on imaging and exam. Bone cysts were histologically distinct from other cyst types in that they lacked epithelium. Histopathologic description of these lesions may include mention of a reactive tissue layer with multinucleate giant cells; however, absence of epithelium truly differentiates bone cysts from dentigerous and radicular cysts. Treatment consisted of aggressive surgical debridement of the cystic structures in 94% of cases. The success rate for surgical treatment was 64.7% with no recurrence noted in 11/17 cases. Two cases lacked sufficient follow up to determine recurrence. This surgical approach resulted in a surgical and postoperative complication rate of 35.3% and recurrence rate of 23.5%.

Diagnosis and treatment of cystic and pseudocystic lesions of the equine maxilla and mandible can result in significant morbidity for the patient, which is often due to the advanced state of the lesions upon initial diagnosis. Clinical signs most commonly associated with these cystic masses included facial swelling (76.5%) and nasal discharge and respiratory issues (both 23.5%). The clinical signs of facial swelling and nasal discharge were similar to other space occupying masses of the equine head such as sinus cysts and neoplasia. Fenner et al. reported facial swelling (67.6%) and nasal discharge (83.7%) in 37 horses examined for paranasal sinus cysts (23). Missing or unerupted teeth in combination with facial swelling were present in all the DTC cases. Differential diagnoses for facial swelling in the presence of missing or unerupted teeth include cystic ameloblastoma or odontoma, but the presence of an unerupted tooth within a cystic structure on diagnostic imaging is strongly suggestive of a dentigerous cyst (7).

On diagnostic imaging, lesions were identified as predominately multiloculated with increased soft tissue opacity (both 82.4%). On radiographs and CT, this change in radiopacity/attenuation indicated the presence of either soft tissue or a uniform fluid. Aspiration or surgical penetration into the site was the main way to determine which was present. Tooth involvement was identified in 94.1% of cases. The only case that did not have dental involvement was a bone cyst of the mandibular body that did not contact the apices of the mandibular premolar and molar teeth. Rarefication of the surrounding bone was the most common bony change, and it occurred in 76.5% of cases. This was expected due to the expansile and benign behavior of cystic masses. CT findings associated with equine paranasal sinus cysts reported 100% cancellous and 50% cortical bone loss adjacent to sinus cysts in 8 horses (24). A retrospective study assessing the clinical presentation, CT findings and outcome of equine odontogenic tumors in 11 horses found that all tumors were associated with maxillary/mandibular bone expansion, alveolar and cortical bone lysis, and cortical bone thinning (25). Cortical bone thickening was observed in 8/11 horses (72.7%) with odontogenic tumors compared to osteosclerosis of adjacent bone in 7/17 (41.2%) cases with non-neoplastic masses in the present study (25). Periosteal proliferation also occurred commonly in 7/11 horses (63.6%) with odontogenic tumors. Periosteal proliferation (documented as sunburst reaction in this study) was observed in a single case of a mandibular body dentigerous cyst associated with an unerupted left mandibular fourth premolar tooth diagnosed in an 18-year-old Warmblood gelding. Further studies with larger case numbers will be needed to determine the association of periosteal reaction with cystic masses.

Six dentigerous cysts were diagnosed in the present study based on the presence of an epithelial lining on histopathology and unerupted or missing teeth on diagnostic imaging and oral exam. Detailed histopathologic descriptions were present for 4/6 DTCs among which the epithelial lining ranged from stratified squamous (both keratinized and non-keratinized) and respiratory epithelium. As the type of epithelium can vary, histopathologists need clinical, oral exam, and diagnostic imaging details to finalize a diagnosis of DTC.

Six bone cysts were diagnosed in the present study. All bone cyst lesions were characterized by reactive bone and fibrovascular tissue bordering the lumen with macrophages and multi-nucleate giant cells and/or amorphous mineral deposits. The key defining feature of a bone cyst is the absence of an epithelial lining. For this reason, these lesions are more accurately termed pseudocysts despite the consistent use of cyst in the literature. Bone cysts were previously defined in horses as aneurysmal bone cysts (ABCs). The term ABC was defined by the 2018 *World Health Organization Classification of Head and Neck Tumours* as “a cystic or multi-cystic expansile osteolytic neoplasm composed of blood-filled spaces separated by fibrous septa containing osteoclast-type giant cells (8).” The second type of bone cyst recognized by the 2018 *WHO Classification of Head and Neck Tumours* was a “simple bone cyst” (SBC) which was defined as “an intraosseous cavity which is devoid of an epithelial lining and is either empty or filled with serous or sanguineous fluid (8).” Due to discrepancies between human and veterinary literature, significant overlap in diagnostic features, and the growing evidence that ABCs in humans may be linked to specific genetic translocation in affected individuals, an attempt was not made to differentiate between ABCs or SBCs in this study (7). The authors propose that the term bone cyst may be utilized to encompass all types of cavitated, pseudocystic intraosseous lesions identified in this study.

Proposed etiologies for bone cyst development include malformation of intraosseous vasculature, trauma, coagulopathy, hematoma development, or underlying neoplasia (7). Bone cysts may be categorized as either primary lesions associated with vascular anomalies or secondary due to trauma, neoplasia, or fibrous dysplasia (5). No inciting cause was identified for the cases in the present study. The ages of the 6 horses affected by bone cysts in the present study ranged from 0.75 years to 24 years with a median age of 8.83 years. Before this study, the largest case series of maxillomandibular equine bone cysts was described by Spoormaker in 2023 and consisted of 3 horses and 1 pony aged 1–22 years (4). These two studies demonstrate that bone cysts can occur in older horses. A differential diagnosis for bone cysts should include neoplasia due to the quickly expansile and multiloculated nature of these masses.

Three radicular cysts were diagnosed in the present study. Two were in the caudal maxilla with sinus involvement, and one was in the rostral maxilla associated with the right maxillary second and third premolar teeth. The diseased teeth identified as the source for these RCs included a dysplastic, non-vital right maxillary second premolar tooth (Case 5), a non-vital/infected left maxillary second molar tooth with an associated periapical abscess (Case 11), and an infected ± non-vital left maxillary first molar tooth (Case 12) with a complicated crown fracture. The paradental chronic inflammation caused by these teeth likely stimulated RC formation. The cystic lining of the two caudal maxillary cases with sinus involvement had respiratory epithelium, while the cystic lining of the rostral maxillary lesion had stratified squamous epithelium. Without a thorough oral exam and dental imaging, it would have been easy to misdiagnose these lesions as paranasal sinus cysts.

Radicular cysts are defined as inflammatory epithelial odontogenic cysts associated with the reserve crown and/or root of a tooth (7). These lesions are typically slow growing and form secondary to chronic inflammation associated with a non-vital and/or endodontically compromised tooth. Radicular cysts are infrequently reported in the veterinary literature and have been previously reported in dogs and an alpaca (13, 15). A single case report of an equine mandibular radicular cyst treated successfully via surgical enucleation exists in the literature (14). It is likely that these lesions are underreported and may be misdiagnosed as equine paranasal sinus cysts. Treatment of these lesions involves surgical debridement of the cyst and extraction of the diseased tooth. If the RC is misdiagnosed and the association with a diseased tooth unrecognized, then the cyst will likely recur.

Two cases of UILs with a radiolucent, cystic appearance on diagnostic imaging were reported in the present study due to the inability to identify a clear etiology on histopathology, oral exam, and diagnostic imaging. No unerupted or primarily diseased teeth were associated with these lesions although teeth were displaced due to the expansile nature of the lesions. The UILs were characterized as unilocular, osteolytic lesions with an interior soft tissue/fluid radiopacity that affected the mandibular incisor and canine teeth. Histopathology of the cavitary lining revealed granulation tissue, fibrosis, and chronic inflammation. If epithelium was identified, it was not clear whether this was true cyst lining or down-growth of surface epithelium, resulting in differentials of an epithelialized periapical abscess vs. severely inflamed cyst. A rostral mandibulectomy was performed to resect the entire lesion in Case 12 due to concern for a potential sarcoma based on the preliminary histopathology. Following additional histopathology of the lesion and collaboration between the clinician and pathologist, a neoplasm was not identified yet definitive diagnosis remained unclear. Lesions similar to these UILs have not been previously reported in the equine literature.

Incisional and excisional biopsy techniques were performed to obtain tissue samples for histopathological diagnosis. Preoperative incisional biopsies (5) were successful at obtaining a diagnosis of a cystic mass in 60% of cases. Excisional biopsies (16) performed at the time of definitive surgery provided definitive diagnoses in 88% of cases (excludes two cases of UIL). Excisional biopsy samples result in larger and more representative samples for histopathologic evaluation and better preservation of lesion anatomy. The authors recommend obtaining surgical access to these cystic and pseudocystic lesions to obtain adequate tissue samples for histopathology.

In the present study, 94% of cases were treated with aggressive surgical debridement of the cystic structure and lining with preservation of surrounding healthy cortical/cancellous bone. A rostral mandibulectomy was performed in one case. When an unerupted or diseased tooth was associated with the lesion, it was extracted. Typically, surgical debridement involves the removal of portions of cystic structures, lining, and suspect tissues until the region is deemed free of unhealthy tissue. Care is taken to preserve critical neurovascular structures and adjacent healthy teeth and bone. Various treatment options for equine maxillary and mandibular cystic masses have been reported with surgical enucleation as the sole treatment being the most common. Surgical enucleation was described for a mandibular radicular cyst (14), paranasal sinus cysts (23, 26), and bone cysts (5, 27). Surgical enucleation combined with corticocancellous bone grafting has been described to successfully treat a case of a mandibular bone cyst in a 1-year-old horse (28). Surgical enucleation combined with collagen hydroxyapatite scaffold implantation has also been described to treat a large, multiloculated mandibular bone cyst in a Thoroughbred filly (2). A combination of surgical enucleation and chemical debridement with 10% formalin was described to successfully treat two cases of maxillary bone cysts (6). Perez et al. described a novel therapy for treating a bilateral rostral mandibular aneurysmal bone cyst in a 1-year-old Standardbred filly consisting of percutaneous doxycycline sclerotherapy (3). Spoormakers et al. reported 4 cases treated with a combination of autologous bone marrow and *β*-tricalcium phosphate with success in 3 cases (4). In people, a large study evaluating treatment of 109 ABCs found a 90.8% success rate for treatment via excision and curettage (*n* = 109, 90.8%, *p* < 0.05). The recurrence rate was 9.2% (11/109 patients) was reported during a 1–45 year follow up period (29). Cases that recurred were treated via repeat curettage, resection or open packing. Such high success rates in humans with surgical management alone may indicate that surgical debridement is an appropriate treatment in other species.

The present study evaluated a diverse grouping of equine maxillomandibular cystic and pseudocystic masses treated with aggressive surgical debridement or mandibulectomy. Follow up ranged from 0 to 872 days postoperatively with a median of 178 days. Only two cases with mandibular body bone cysts (case 4 and 11) were considered lost to follow-up due to a lack or limited amount of time to follow-up. Sixty-six percent of cases with follow-up had no documented recurrence. The lesion recurrence rate after surgical treatment was 23.5%. The difference in recurrence rates after surgical debridement between human studies and the present study may be due to the complicated anatomy of the equine paranasal sinus system limiting the ability of the surgeon to completely debride the affected region, and diagnosis of these lesions in horses when they are at an advanced state. Lesion recurrence is likely due to remnant cystic lining or tissue within the surgical site due to inaccessible anatomic regions during surgery or the wrapping of cystic tissue around neurovascular structures and other critical anatomy. Therefore, radiographic rechecks should be recommended every 6 months for 3–4 years at a minimum to monitor for recurrence. Owners should be counseled ahead of surgery that repeated debridement may be necessary to achieve resolution if recurrence is detected.

Long term resolution following surgical excision of paranasal sinus cysts has been reported to range from 78.6% (23) to 82% (26). The overall success rate of 64.7% in the present study may have been impacted by the lack of adequate follow-up for 2 cases and the fact that 2 cases (case 5 and 17) did not have definitive treatment with the first surgery. The treatment plan for case 5, a RC, included two surgeries to avoid the creation of a large oronasal fistula. The RC was extraorally aggressively debrided in the first procedure and allowed to heal. Once soft tissue had filled the RC defect, the associated diseased second premolar tooth was extracted intraorally. A third surgery was required roughly 2 years later to remove a large concrescence of reactive nodular hypercementosis surrounded by a cystic structure. Case 17 involved DTCs surrounding many premolar and molar teeth in a young pony. This pony was eventually euthanized following development of a large oroantral fistula and airway reduction on the contralateral maxilla to the initial surgery.

The surgical and postoperative complication rate in this study was 35.3% with 1 case in the rostral maxilla, 2 cases located in the caudal maxilla/sinus, and 3 cases in the body of the mandible. The rostral maxillary complication of temporary facial nerve paresis occurred secondary to intraoperative trauma to the facial nerve and resolved postoperatively. Caudal maxillary complications included oroantral fistula, sinus flap suture reaction, and sinus flap mycosis. Mandibular body complications included bony sequestrum, orofacial fistulas, and a single case of an intraoperative iatrogenic mandibular ramus fracture. Complication rates following sinus flap surgery have been previously reported (23, 26, 30). In Fenner et al.’s (23) study assessing complications related to the surgical treatment of 37 paranasal sinus cysts, 32.4% developed surgical site infections, 16.6% developed nasofrontal suture periostitis, and 13.5% developed mycotic infection of mucosal tissue. A recent study evaluating treatment of 229 cases of equine paranasal sinus disease via trephination found that complications including suture line periostitis and fistula formation occurred in 46% of all cases and recurrence of disease occurred in 36.7% of cases (30). These complication rates are comparable to the present study and indicate that invasive surgical treatment of diseases of the equine head is associated with surgical morbidity.

Limitations to this study included non-standardized medical records, histopathology reports from various pathologists, multiple surgeons, variable follow-up, and small sample size. As cases were recruited from six different practices, variability in medical record keeping, personnel, and laboratories could not be avoided. Although this is the largest study of equine cystic and pseudocystic maxillomandibular masses to the author’s knowledge, the case numbers for each cyst type were too few to assess statistical significance.

## Conclusion

Cystic and pseudocystic masses of the equine maxilla and mandible result in clinically significant morbidity for the equine patient of a wide range of ages. These lesions commonly result in facial swellings that are often associated with missing or unerupted teeth. On diagnostic imaging, the majority of lesions are well-demarcated and multilocular with a soft tissue/fluid radiopacity and rarefication of the surrounding bone. Identification of primary dental involvement, either in the form of unerupted or diseased teeth, can greatly aid in diagnosis. Definitive diagnosis is most accurately obtained by performing an excisional biopsy at the time of definitive surgery. The submission of all involved tissue types (cystic, bone, and dental) for histopathology along with the inclusion of history, diagnostic imaging, and oral exam findings significantly aid the pathologist in determining an accurate diagnosis. On histopathology, dentigerous and radicular cysts have an epithelial lining while bone cysts do not. Despite the relative success of aggressive surgical debridement, recurrence is possible; therefore, frequent postoperative radiographic rechecks are recommended.

## Data Availability

The raw data supporting the conclusions of this article will be made available by the authors, without undue reservation.
